# Speech and language therapy assessment based on applied behavior analysis: a scoping review

**DOI:** 10.1590/2317-1782/e20240155en

**Published:** 2025-08-11

**Authors:** Débora Pontes Cavalcante Almeida, Cíntia Alves Salgado Azoni, Larissa Nadjara Alves Almeida, Ivonaldo Leidson Barbosa Lima, Isabelle Cahino Delgado

**Affiliations:** 1 Programa de Pós-graduação em Fonoaudiologia, Universidade Federal da Paraíba – UFPB - João Pessoa (PB), Brasil.; 2 Departamento de Fonoaudiologia, Universidade do Rio Grande do Norte – UFRN - Natal (RN), Brasil.; 3 Departamento de Fonoaudiologia, Universidade Federal da Paraíba – UFPB - João Pessoa (PB), Brasil.

**Keywords:** Autistic Spectrum Disorder, Applied Behavior Analysis, Language Assessment, Scoping Review, Language and Hearing Sciences

## Abstract

**Purpose:**

To identify and describe language assessment protocols from the perspective of ABA used in speech-language therapy.

**Research strategies:**

The question that guided the study was: What are the ABA-based language assessment protocols currently used in speech therapy? For the electronic search of articles, the databases used were Medline/PubMed, Lilacs, Web of Science and Scopus. The search strategy used indexed and free uniterms related to PCC.

**Selection criteria:**

Studies with a cross-sectional design were included, considering individuals undergoing language assessment as the population; ABA-based assessment protocols in speech-language therapy were included. Studies that did not focus on language assessment and with a literature review design, letters to the editor, books, abstracts from proceedings, opinion articles and technical articles were excluded.

**Data analysis:**

The data were analyzed descriptively, analyzing the studies' levels of evidence.

**Results:**

A total of 6,859 articles were identified in all the databases. Of these, 17 articles were selected by title, after reading the abstract. 12 were excluded because they did not meet the inclusion criteria: 4 were review articles, 6 did not fit the objective of this research and 2 were repeated between the databases. Five were then selected for full text reading and subsequent data analysis.

**Conclusion:**

Only two language assessment protocols from the perspective of ABA used in speech therapy were found in this review: VB-MAPP and ABLLS-R. Both do not have validation studies for Brazilian Portuguese

## INTRODUCTION

Applied Behavior Analysis, commonly referred to as ABA, is a scientific approach that focuses on understanding and improving human behavior^(1)^. Grounded in decades of research, ABA stands out for its solid empirical base and individualized approaches to modifying behaviors. Essentially, ABA uses learning principles and techniques to address a wide range of social, communicative, behavioral and learning skills in individuals with diverse needs^(2)^.

The ABA is based on the concept that behavior is influenced and modified by the environment and that meaningful and lasting learning can be achieved through strategic changes in this environment^(3)^. This involves the systematic use of reinforcement techniques to increase desirable behaviors and reduce undesirable behaviors. ABA is particularly effective in working with individuals with Autistic Spectrum Disorders (ASD), but its applicability extends to a variety of contexts and populations, including schools, clinics, homes, and workplaces^(4)^.

Within the range of professionals dedicated to the treatment and support of individuals with Autism Spectrum Disorder (ASD), speech therapists play a crucial role, using strategies derived from ABA science to optimize the effectiveness of their interventions^(5)^. The integration of ABA in speech therapy practice allows a more structured and data-driven approach to developing communication and language skills, essential for individuals with ASD. This synergy is manifested in the application of specific behavioral techniques, such as positive reinforcement, functional behavior analysis and generalization training, aimed at improving communication and reducing challenging behaviors^(3)^.

By using the principles of ABA, speech therapists can create personalized treatment plans focused on the unique needs of each individual with ASD, promoting measurable and significant progress in their communication and social interaction skills. This integrated approach not only enriches the clinical practice of speech therapy, but also contributes substantially to the quality of life and development of individuals with ASD^(6)^.

ABA science is highly customizable, allowing intervention plans to be tailored to individual needs, strengths, skills and interests. This customized approach is fundamental to the effectiveness of ABA, as it recognizes that there is no one-size-fits-all solution when it comes to human development and learning. In addition, continuous analysis and data evaluation are critical components in ABA practice, ensuring that interventions are guided by observable progress and measurable results^(7)^.

Evaluation is a fundamental pillar in ABA, serving as the starting point for any effective behavioral intervention. This meticulous and scientifically-oriented process aims to understand the nuances of individual behavior, its functions and the environmental factors that influence it. The ABA assessment is not only a means to identify areas of need, but also a tool to establish personalized and measurable therapeutic goals^(6)^.

Assessment in ABA focuses on the collection and analysis of detailed behavioral data, performed through a variety of techniques such as direct observations, interviews with people close to the child (for example, parents, caregivers, teachers), and the application of standardized protocols. These methods are used to create a comprehensive behavioral profile of the individual, which will project the formulation of hypotheses about the functions of behaviors and the selection of intervention strategies^(2,4)^.

The implementation of standardized evaluation protocols, such as VB-MAPP and ABLLS-R, within the scope of ABA is an essential practice for the diagnosis and planning of effective behavioral interventions. These protocols should be designed to provide a comprehensive and systematic understanding of the individual’s behavior, his skills and challenges within a well-established theoretical framework^(6,8)^.

The speech-language assessment in children with Autistic Spectrum Disorder (ASD) represents a unique and multifaceted challenge, requiring the integration of several tools and approaches for a holistic understanding of the child’s needs. This complexity is due in large part to the diverse nature of ASD, which affects communication and behavior in various ways. In this context, the ABA appears as an essential component in the speech therapist’s toolbox, providing a framework to assess and address communication difficulties more effectively^(8)^.

However, the application of ABA protocols, especially those originated in international literature, such as VB-MAPP and ABLLS-R, presents significant challenges in Brazilian clinical practice. The operationalization of these protocols often requires specialized training in ABA and faces obstacles such as the lack of validation for the Brazilian population, which can limit their applicability and effectiveness. This scenario highlights the need to adapt or develop new instruments and evaluation methods that are culturally sensitive and appropriate to the Brazilian context^(4)^.

The importance of accurate assessment in the treatment of children with ASD cannot be underestimated. An inadequate evaluation can lead to failures in the planning and implementation of treatment, negatively affecting the prognosis and results for the patient. Therefore, it is crucial that speech-language assessment in children with ASD be comprehensive, meticulous and tailored to their individual needs. This introduction aims to highlight the relevance of integrative and culturally adapted assessment approaches in speech therapy for children with ASD, emphasizing the need for evidence-based and context-sensitive practice to ensure the effectiveness of interventions^(1,6)^.

Given the growing importance of language assessment protocols in speech therapy, it is essential to understand the quality of the measures used and evaluate their adequacy and effectiveness in clinical practice. These protocols are vital to address communication and behavior needs in a variety of clinical contexts.

Therefore, the objective of this scope review was to identify and describe the language evaluation protocols under the ABA perspective used in speech therapy. This review aims to provide a comprehensive understanding of existing protocols, highlighting best practices and identifying possible gaps in the literature, to improve the quality of assessment and language intervention in speech therapy.

## METHODS

The proposed scoping review was carried out according to the JBI methodology and based on the Preferred Reporting Items for Systematic Reviews and Meta-Analyses (PRISMA) recommendations. The protocol was registered with the OSF INSTITUTIONS, under number: 10.17605/OSF.IO/D2FMV.

### Research strategy

To conduct this review, the following research question was adopted: What are and how are the ABA-based language assessment protocols currently used in Speech Therapy?

The criteria for search strategy were adapted from the acronym PCC: Population (P) - individuals who will undergo language assessment; Concept (C) - Effectiveness, efficiency and applicability of evaluation protocols based on ABA; Context (C) - elaboration and validation of language evaluation protocols based on Applied Behavior Analysis (ABA).

The search was carried out on January 10, 2024. We performed electronic searches in the databases LILACS, PubMed/Medline, Scopus and Web of Science, using a combination of descriptors and free terms (Chart 1). The references of the selected studies were also included.

**Chart 1:** Search strategy

**Database**	**Search Strategy**
LILACS	(“autism spectrum disorder” OR “autism” OR Children) AND (“language assessment” OR “validation study” OR “protocol” OR “language test” OR “assessment”) AND (“verbal behavior” OR “applied behavior analysis”)
PUBMED	(“autism spectrum disorder”[All Fields] OR “autism”[All Fields] OR “children”[All Fields]) AND (“language assessment”[All Fields] OR “validation study”[All Fields] OR “protocol”[All Fields] OR “language test” [All Fields] OR “assessment”[All Fields]) AND (“verbal behavior” [All Fields] OR “applied behavior analysis”[All Fields])
SCOPUS	(TITLE-ABS-KEY ((“autism spectrum disorder” OR “autism” OR “children”)) AND TITLE-ABS-KEY ((“language assessment” OR “validation study” OR “protocol” OR “language test” OR “assessment”)) AND TITLE-ABS-KEY ((“verbal behavior” OR “applied behavior analysis”)))

The search in the databases was conducted by two researchers independently using the Rayyan tool, after an initial calibration for selection of articles. The material analysis was carried out in sequential steps. In the first stage, the articles were selected based on their titles, taking into account the proposed theme. In the second stage, abstracts of articles were read to exclude those that did not meet the eligibility criteria. In the third stage, the articles that fit the objectives of the study were read in full. In the fourth step, duplicate references found in the databases consulted were eliminated. In case of disagreement among the evaluators, a third-party evaluator was consulted to resolve the disagreement and decide whether the articles in question would be included or excluded. Then, the articles were analyzed according to the observation of variables such as: authors, year, database, country, journal, objective and outcome. The data were tabulated in Microsoft Excel digital spreadsheet for further analysis, interpretation and construction of descriptive tables.

The data were analyzed in a descriptive way, by means of absolute and relative frequency, as well as the nominal description of the variables in demonstrative frames, considering the number of studies selected, their profile, and levels of evidence that do not meet the requirements for conducting meta-analysis, highlighting the need for research on the subject.

### Selection criteria:

We included studies that investigated the validation process and the application of ABA-based assessment protocols in speech therapy; aimed at children; focusing on language; containing information about psychometric properties, such as reliability and accuracy. There was no limit of year and language. We excluded studies that did not use ABA-based evaluation protocols, that did not focus on language evaluation, with literature review design, letters to the editor, books, abstracts of proceedings of scientific events, opinion articles and technical articles.

### Data analysis:

The articles were analyzed according to the observation of variables such as authors, year, database, country, journal, objective and outcome. The data were tabulated in Microsoft Excel digital spreadsheet for further analysis, interpretation and construction of descriptive charts.

The data were analyzed in a descriptive way, by means of absolute and relative frequency, as well as the nominal description of the variables in demonstrative frames, considering the number of studies selected, their profile, and levels of evidence that do not meet the requirements for conducting meta-analysis, highlighting the need for research on the subject.

The certainty of the evidence was analyzed considering the measurement properties and methodological quality, applying levels of evidence. This method will allow a comprehensive and detailed mapping of ABA assessment protocols in speech therapy, contributing to the optimization of clinical practices in this area.

## RESULTS

**Figure 1:**
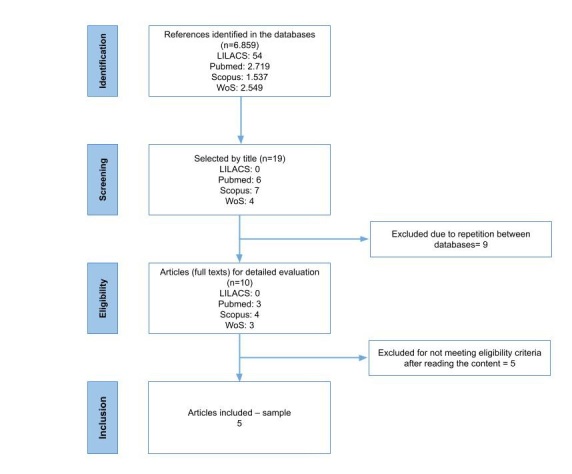
Study selection strategy flowchart

A total of 6,859 articles were identified in all databases, 54 in LILACS, 2,719 in PubMed/Medline, 1,537 in Scopus and 2,549 in the Web of Science (WoS). Of these, 17 articles were selected by title, after reading the abstract, 12 were excluded because they did not meet the inclusion criteria: 4 were review articles, 6 did not suit the objective of this research and 2 were repeated among the databases. In sequence, 5 were selected for a complete reading of the text and subsequent analysis of the data, according to the flow chart shown in Figure 1.

Charts 2 and 3 show the main findings of the variables literature regarding the general characteristics of the studies, their objective and outcome, respectively.

**Chart 2:** General characteristics of studies that performed language assessment based on ABA, selected according to eligibility criteria

ARTICLES	AUTHOR	DATABASE	COUNTRY	JOURNAL
1	Padilla et al.^(9)^	*PubMed*	United States	*Journal of Autism and Developmental Disorders*
2	Barnes et al.^(10)^	*Scopus*	United States	*Analysis Verbal Behav*
3	Sundberg and Michael^(7)^	*Scopus/WoS*	United States	*Behavior Modification*
4	Montallana et al.^(11)^	*PubMed/Scopus*	United States	*Journal of Autism and Developmental Disorders*
5	Myers et al.^(8)^	*WoS*	United States	*Journal of Autism and Developmental Disorders*

Of the articles presented, there was publication between 2001 and 2022, with an increase in the frequency of publications from 2019, (60.0%; n=3) were indexed in the Scopus database, (100.0%; n=5) are studies conducted in the United States, and (60.0%; n=3) were published in the Journal of Autism and Developmental Disorders (Chart 2).

Most studies (n=3; 60%) of the studies addressed the use of the Verbal Behavior Milestones Assessment and Placement Program (VB-MAPP), with two referring to the validation process and another to its applicability. Sundberg and Michael^(7)^ discussed the evaluation proposed by Skinner, pointing out the importance of its dimensions to support ASD evaluation. We also found a study that used the Evaluation of Basic Language and Learning Skills - Revised (ABLLS-R), which was able to monitor the development trajectory of the child (Chart 3).

**Chart 3:** Description of findings regarding ABA-based language assessment in selected studies

**ARTICLE**	**VARIABLE**	**RESULT**
**1**	INSTRUMENT	Verbal Behavior Milestones Assessment and Placement Program (VB-MAPP)
	OBJECTIVE	To assess the content validity of the VB-MAPP, including the Early Echoic Skills Assessment (EESA) and the Barriers Assessment
	OUTCOME	Content validity was moderate to strong, although some areas had limited or conflicting support.
**2**	INSTRUMENT	Verbal Behavior Milestones Assessment and Placement Program (VB-MAPP)
	OBJECTIVE	To examine the effects of behavioral skills training on the administration of the VB-MAPP milestone assessment by education professionals.
	OUTCOME	The training resulted in immediate increases in professional performance
**3**	INSTRUMENT	Skinner Analysis
	OBJECTIVE	To analyze the importance of Skinner analysis of verbal behavior in the treatment of children with autism
	OUTCOME	Skinner's Verbal Behavior Analysis can improve intervention programs for children with autism who already benefit from Applied Behavior Analysis (ABA)
**4**	INSTRUMENT	Verbal Behavior Milestones Assessment and Placement Program (VB-MAPP)
	OBJECTIVE	To explore inter-rater consistency in the application of VB-MAPP
	OUTCOME	Although the Milestones Assessment and Barriers Assessment showed good and moderate reliability, respectively, the individual domains of each assessment were less reliable.
**5**	INSTRUMENT	Assessment of Basic Language and Learning Skills – Revised (ABLLS-R)
	OBJECTIVE	To evaluate the effectiveness of a state Medicaid program that provides home-based Early Intensive Behavioral Intervention services to children with Autism Spectrum Disorder.
	OUTCOME	There was significant variation in the initial scores of the children's ABLLS-R and its growth trajectories, with differences based on the initial age, ethnicity and geographical location

## DISCUSSION

Language assessment is the basis for clinical decisions. The information collected directs the strategies that will be used throughout the intervention, so the data collected must be functional and effective. Well-structured, validated and responsive evaluation instruments support the evaluation process and enable a more reliable and evidence-based decision-making. Therefore, it is important to research, know and select the best assessment instruments, both in clinical and research.

The adoption of effective language evaluation protocols allows the standardization of collected information, replicability by other professionals and institutions, a reliable monitoring and reassessment, and consequently facilitates communication between clinical professionals and researchers, and even school. These aspects are important for individuals diagnosed with ASD, long-term accompanied by different professionals.

Despite this, it was observed during the searches that standardized and validated language evaluation tools in ASD are scarce^(12)^. There is a need for more investment in the validation processes and psychometric measures, especially when it concerns the interface language and ABA, given the results presented in this work, which found low amount of research on the subject, with only two protocols described, not yet validated for the Brazilian population according to the recommended, highlighting the need for studies focused on the proposal and validation of instruments aimed at language evaluation in ASD and based on Applied Behavior Analysis.

ABA plays a vital role in the development of language evaluation protocols, especially in the context of ASD. The effectiveness of these protocols lies in their ability to provide a detailed understanding of an individual’s language and communication skills, allowing personalized and focused interventions. This article reviews the existing literature on language assessment protocols in ABA, highlighting innovative approaches and evidence-based practices^(1)^.

The VB-MAPP (Verbal Behavior Milestones Assessment and Placement Program) is one of the protocols presented in the selected studies of this review, based on the analysis of Skinner’s developed to assess children with autism or other developmental disorders. It focuses on several areas, including language milestones, learning skills and social behavior. The program is divided into different levels and includes assessments such as initial echoic skills (EESA) and a barrier assessment, which identifies barriers to learning and communication. The VB-MAPP evaluates both expression and understanding of language, providing a comprehensive picture of the child’s linguistic development^(9)^.

It is divided into several sections, such as the assessment of milestones: it evaluates 170 milestones of language development and learning, divided into 3 levels according to the age of the child. Includes aspects such as command (request), touch (naming), echoic (vocal imitation), intraverbal (conversation), group skills, social play, among others; evaluation of barriers: identifies and evaluates barriers to learning, such as problematic behaviors, poor echoic skills, lack of motivation, among others. This assessment helps to understand what is preventing the child from learning effectively; Initial Echoic Skills Assessment (EESA): focuses on assessing the child’s ability to imitate sounds and words, which is fundamental for speech development; Transition assessment: aimed at children who are ready to move from a structured learning environment to a more natural one, such as a regular classroom and placement planning and teaching (TASK): provides guidelines for the development of individualized teaching plans, based on information collected in previous evaluations^(9)^.

Article 1, “Evidence of Content Validity for the Verbal Behavior Milestones Assessment and Placement Program (VB-MAPP)”, investigates the content validity of the VB-MAPP. The survey involved a panel of experts who evaluated several aspects of the program, including relevance, age adequacy and measurement methods^(9)^.

The validity of content is a psychometric process recommended by the validation guidelines that observes the consistency and adequacy of the instrument with respect to the topic, writing, format, tasks or questions of a test, application response instructions. the elaboration of the items, considering syntactic and semantic aspects that contribute to the clarity, relevance, coherence and comprehensiveness of the items, in addition to the operational aspects^(13)^. The instrument should be evaluated by experts, specialists in the thematic area of the instrument, who mainly evaluate the representativeness and relevance of the items in relation to the proposed outcome, through the calculation of the general Content Validity Index (CVI) and by Item (CVI-I) for presentation score of agreement between the judges^(13)^.

In the results of this research, the effectiveness of this protocol in the evaluation of linguistic and communicative skills is highlighted. The results also indicated a moderate to strong content validity for VB-MAPP, although some domains had limited or conflicting support. This suggests that VB-MAPP is a useful tool, but some aspects may need refinement. The study also addresses the importance of continuous and adaptive assessments, essential to monitor progress and adjust educational and therapeutic interventions for children with autism^(9)^.

Still focusing on VB-MAPP, the article by Barnes et al.^(10)^, “Implementing the Verbal Behavior Milestones Assessment and Placement Program (VB-MAPP): Teaching Assessment Techniques” examines the impact of behavioral skills training (BST) on the administration of VB-MAPP by education professionals. The research demonstrated that BST resulted in immediate improvements in participants' performance in applying the VB-MAPP.

The focus on the importance of proper training for effective administration of VB-MAPP, the research reinforces the idea that well-trained professionals are crucial to ensure accurate and effective assessments, essential in the development of individualized intervention plans for children with autism. The study also suggests that competent application of VB-MAPP can provide deeper insights into children’s language and learning needs, directing more effective interventions^(10)^.

There is a complexity of language assessment in children with autism and the results of this research highlight the need for a deep understanding of the VB-MAPP protocol for an effective application. The results also show immediate improvements in the ability of education professionals to administer VB-MAPP after BST training, suggesting that targeted training can increase the accuracy and effectiveness of assessments. This finding has significant implications for the training and professional development of educators and therapists working with children in the autistic spectrum, emphasizing the need for robust and evidence-based training programs^(10)^.

It is known that factors related to the language, culture, environment and context of application, characteristics of the application, such as interferences of the applicator, type of responses, objective or subjective, answer key, instrument size, application model, interview or self-application, can directly interfere in the collection of the assessment^(14)^. Therefore, it is important to define these aspects, as well as training for the professionals who will make the application, as carried out in the aforementioned protocol.

The VB-MAPP passed through other validation steps. Montallana et al.^(11)^ aimed to analyze its interevaluators reliability of the VB-MAPP. The research revealed that, although the VB-MAPP Mark Assessment and Barrier Assessment presented good and moderate reliability respectively, the reliability in the individual domains was less consistent.

The variability in reliability reinforces the need for additional training for evaluators and possible refinements in the VB-MAPP protocol. This discussion highlights the importance of ensuring consistency and accuracy in assessments, which are fundamental to developing effective educational and therapeutic interventions for children with autism. The research also points to the need for additional studies to explore the causes of variability in reliability and to optimize the use of VB-MAPP in different contexts^(11)^.

Different professionals may interpret and score the VB-MAPP inconsistently. This may have significant implications in the formulation of educational and therapeutic plans for children with autism. These results emphasize the need for more research to improve the reliability and effectiveness of VB-MAPP^(11)^.

The third study, entitled “The Benefits of Skinner’s Analysis of Verbal Behavior for Children With Autism” by Mark L. Sundberg and Jack Michael^(7)^, is a theoretical discussion on the application of Skinner’s verbal behavior analysis in the context of autism. The article suggests that this approach can significantly enrich interventions for children with autism, emphasizing the importance of understanding and teaching different verbal operators in a specific and targeted way.

The analysis of verbal behavior by B.F. Skinner is a significant approach in the study of language, Skinner proposes that verbal behavior should be understood as a function of operant behavior, where language is shaped and maintained by the environment. It identifies different types of verbal operants, such as command (request), touch (naming), echoic (vocal imitation), and intraverbal (verbal responses that are not echoes of heard words). This approach focuses on the practical function of language and not just its form or structure, allowing a deeper understanding of how children with autism learn and use language in different contexts^(7)^.

Skinner’s analysis explores how the analysis of verbal behavior offers a detailed and functional perspective on language, differentiating itself from approaches that focus only on the form and structure of language. By understanding the role of language in the context of behavior, therapists and educators can create more effective intervention programs that address the specific communication needs of each child with autism. This may include adapting methods to develop functional communication skills and considering factors such as motivation and reinforcement in language learning^(7)^.

The last article found in this review, “Analysis of a Statewide Early Intervention Program for Young Children with Autism Spectrum Disorder,” analyzes the effectiveness of an early intervention program for children with autism in a rural USA state. The study used the Assessment of Basic Language and Learning Skills - Revised (ABLLS-R) to evaluate children^(8)^.

The ABLLS-R is a comprehensive assessment and curriculum tool for children with autism and other developmental disorders. It assesses a wide range of language and learning skills, including communication, social skills, self-care, motor skills and academic. The ABLLS-R is designed to identify specific disabilities and develop customized educational programs based on ABA principles. It is a detailed instrument that offers clear guidelines for measuring and improving essential skills^(8)^.

The results of the study indicate significant variations in children’s initial ABLLS-R scores and their growth trajectories, suggesting that factors such as age of program entry, ethnicity and geographic location may influence language development. This highlights the importance of considering these factors when developing and evaluating intervention programs. The discussion also suggests the need for more research to better understand how different variables impact the progress of children with autism in early intervention programs^(8)^.

Moreover, the results point to the importance of considering the socio-cultural and environmental context in the formulation of intervention programs, as well as the need for longitudinal monitoring to evaluate the effectiveness of interventions over time. These findings are vital to inform evidence-based practices and policies for the education and care of children with autism^(8)^.

Not many studies were found that brought evaluation instruments based on ABA to evaluate language. In addition, the studies found do not contemplate the validation of these instruments for Brazilian Portuguese. There is space and the need for more validation studies of language evaluation protocols in children, especially those with ASD.

## CONCLUSION

Only two language assessment protocols under the ABA perspective used in speech therapy were found in this review: VB-MAPP and ABLLS-R. Both have no validation studies for Brazilian Portuguese.

The reviewed articles demonstrate the complexity, importance and scarcity of ABA language assessment protocols such as VB-MAPP and ABLLS-R in intervention for children with autism. They also highlight the need for adequate training for professionals in the administration of these tools and the importance of individualized approaches, as suggested by Skinner’s analysis of verbal behavior. The variability in inter-evaluators reliability of VB-MAPP and the differences in language development trajectories observed by ABLLS-R underline the need for adapted evaluation strategies and continuous research to improve the effectiveness of evaluation protocols, aiming at a more efficient intervention of language in autism.
