# Supplementary material for: Speech and language therapy assessment based on applied behavior analysis: a scoping review
Source: Codas. 2025 Aug 11;37(4):e20240155. doi: 10.1590/2317-1782/e20240155en (PMC12341758; doi:10.1590/2317-1782/e20240155en)
Supplement: Translation

# Translation: Avaliação fonoaudiológica de linguagem baseada em análise do comportamento aplicada: revisão de escopo

**DOI:** 10.1590/2317-1782/e20240155pt

**Authors:** Débora Pontes Cavalcante Almeida, Cíntia Alves Salgado Azoni, Larissa Nadjara Alves Almeida, Ivonaldo Leidson Barbosa Lima, Isabelle Cahino Delgado

**Affiliations:** 1 Programa de Pós-graduação em Fonoaudiologia, Universidade Federal da Paraíba – UFPB - João Pessoa (PB), Brasil.; 2 Departamento de Fonoaudiologia, Universidade do Rio Grande do Norte – UFRN - Natal (RN), Brasil.; 3 Departamento de Fonoaudiologia, Universidade Federal da Paraíba – UFPB - João Pessoa (PB), Brasil.

**Keywords:** Transtorno do Espectro Autista, Análise do Comportamento Aplicada, Avaliação da Linguagem, Revisão de Escopo, Fonoaudiologia

## Abstract

**Objetivo:**

Identificar e descrever os protocolos de avaliação de linguagem sob a perspectiva da ABA utilizados na fonoaudiologia.

**Estratégia de pesquisa:**

A questão que norteou o estudo foi: Quais são os protocolos de avaliação de linguagem baseados em ABA atualmente utilizados na Fonoaudiologia? Para a busca eletrônica dos artigos, as bases de dados utilizadas foram Medline/PubMed, Lilacs, Web of Science e Scopus. A estratégia de busca utilizou unitermos indexados e livres relacionados ao PCC.

**Critérios de seleção:**

Foram incluídos estudos com delineamento transversal, considerando como população indivíduos submetidos a avaliação de linguagem; foram incluídos Protocolos de Avaliação baseados em ABA na Fonoaudiologia. Foram excluídos estudos que não focaram em avaliação de linguagem e com delineamento de revisão de literatura, cartas ao editor, livros, resumos de anais, artigos de opinião e artigos técnicos.

**Análise dos dados:**

Os dados foram analisados de forma descritiva, com análise dos níveis de evidência dos estudos.

**Resultados:**

Foram obtidos 6.859 artigos. Destes, 17 artigos foram selecionados pelo título, após a leitura do resumo. 12 foram excluídos por não cumprirem os critérios de inclusão: 4 eram artigos de revisão, 6 não se adequavam ao objetivo desta pesquisa e 2 estavam repetidos entre as bases de dados. Na sequência 5 foram selecionados para leitura completa do texto e posterior análise dos dados.

**Conclusão:**

Apenas dois protocolos de avaliação de linguagem sob a perspectiva da ABA utilizados na fonoaudiologia foram encontrados nesta revisão: VB-MAPP e o ABLLS-R. Ambos não possuem estudos de validação para o português brasileiro.

## INTRODUÇÃO

A Análise do Comportamento Aplicada, comumente referida pela sigla ABA, do inglês *Applied Behavior Analysis*, é uma abordagem científica que se concentra na compreensão e melhoria do comportamento humano^(1)^. Fundamentada em décadas de pesquisa, a ABA se destaca por sua base empírica sólida e abordagens individualizadas para modificar comportamentos. Essencialmente, a ABA utiliza princípios e técnicas de aprendizagem para abordar uma ampla gama de habilidades sociais, comunicativas, comportamentais e de aprendizagem em indivíduos com diversas necessidades^(2)^.

A ABA embasa-se no conceito de que o comportamento é influenciado e modificado pelo ambiente e que aprendizados significativos e duradouros podem ser alcançados através de mudanças estratégicas nesse ambiente^(3)^. Isso envolve o uso sistemático de técnicas de reforçamento para aumentar comportamentos desejáveis e reduzir comportamentos indesejáveis. A ABA se mostra particularmente eficaz no trabalho com indivíduos com Transtornos do Espectro Autista (TEA), mas sua aplicabilidade se estende a uma variedade de contextos e populações, incluindo escolas, clínicas, lares e locais de trabalho^(4)^.

Dentro da gama de profissionais dedicados ao tratamento e apoio de indivíduos com Transtorno do Espectro Autista (TEA), o fonoaudiólogo desempenha um papel crucial, utilizando estratégias derivadas da ciência ABA para otimizar a eficácia de suas intervenções^(5)^. A integração da ABA na prática fonoaudiológica permite uma abordagem mais estruturada e orientada por dados no desenvolvimento de habilidades de comunicação e linguagem, essenciais para indivíduos com TEA. Esta sinergia se manifesta na aplicação de técnicas comportamentais específicas, como reforço positivo, análise funcional do comportamento e treinamento de generalização, visando melhorar a comunicação e reduzir comportamentos desafiadores^(3)^.

Ao utilizar os princípios da ABA, o fonoaudiólogo pode criar planos de tratamento personalizados, focados nas necessidades únicas de cada indivíduo com TEA, promovendo um progresso mensurável e significativo em suas habilidades de comunicação e interação social. Esta abordagem integrada não só enriquece a prática clínica da Fonoaudiologia, mas também contribui de forma substancial para a qualidade de vida e o desenvolvimento dos indivíduos com TEA^(6)^.

A ciência ABA é altamente personalizável, permitindo que os planos de intervenção sejam adaptados às necessidades individuais, forças, habilidades e interesses do indivíduo. Essa abordagem personalizada é fundamental para a eficácia da ABA, pois reconhece que não existe uma solução única para todos no que se refere ao desenvolvimento humano e à aprendizagem. Além disso, a análise contínua e a avaliação de dados são componentes críticos na prática da ABA, garantindo que as intervenções sejam guiadas por progressos observáveis e resultados mensuráveis^(7)^.

A avaliação representa um pilar fundamental na ABA, servindo como o ponto de partida para qualquer intervenção comportamental eficaz. Este processo meticuloso e cientificamente orientado visa compreender as nuances do comportamento individual, suas funções e os fatores ambientais que o influenciam. A avaliação ABA não é apenas um meio para identificar áreas de necessidade, mas também uma ferramenta para estabelecer objetivos terapêuticos personalizados e mensuráveis^(6)^.

A avaliação em ABA concentra-se na coleta e análise de dados comportamentais detalhados, realizada através de uma variedade de técnicas como observações diretas, entrevistas com pessoas do convívio próximo da criança (por exemplo, pais, cuidadores, professores), e a aplicação de protocolos padronizados. Esses métodos são empregados para criar um perfil comportamental abrangente do indivíduo, o qual projetará a formulação de hipóteses sobre as funções dos comportamentos e a seleção de estratégias de intervenção^(2,4)^.

A implementação de protocolos padronizados de avaliação, como VB-MAPP e ABLLS-R, no âmbito da ABA constitui uma prática essencial para o diagnóstico e planejamento de intervenções comportamentais eficazes. Esses protocolos devem ser desenhados para fornecer um entendimento abrangente e sistemático do comportamento do indivíduo, suas habilidades e desafios, dentro de um quadro teórico bem estabelecido^(6,8)^.

A avaliação fonoaudiológica em crianças com Transtorno do Espectro Autista (TEA) representa um desafio único e multifacetado, exigindo a integração de diversas ferramentas e abordagens para uma compreensão holística das necessidades da criança. Essa complexidade se deve, em grande parte, à natureza diversa do TEA, que afeta a comunicação e o comportamento de maneiras variadas. Neste contexto, a ABA surge como um componente essencial na caixa de ferramentas do fonoaudiólogo, fornecendo uma estrutura para avaliar e abordar as dificuldades de comunicação de forma mais efetiva^(8)^.

Contudo, a aplicação de protocolos em ABA, especialmente aqueles originados na literatura internacional, como o VB-MAPP e ABLLS-R, apresenta desafios significativos na prática clínica brasileira. A operacionalização desses protocolos frequentemente requer uma formação especializada em ABA e enfrenta obstáculos como a falta de validação para a população brasileira, o que pode limitar sua aplicabilidade e eficácia. Este cenário destaca a necessidade de adaptar ou desenvolver novos instrumentos e métodos de avaliação que sejam culturalmente sensíveis e apropriados ao contexto brasileiro^(4)^.

A importância da avaliação precisa no tratamento de crianças com TEA não pode ser subestimada. Uma avaliação inadequada pode levar a falhas no planejamento e implementação do tratamento, afetando negativamente o prognóstico e os resultados para o paciente. Portanto, é crucial que a avaliação fonoaudiológica em crianças com TEA seja abrangente, meticulosa e adaptada às suas necessidades individuais. Esta introdução visa destacar a relevância de abordagens de avaliação integrativas e adaptadas culturalmente na fonoaudiologia para crianças com TEA, enfatizando a necessidade de uma prática baseada em evidências e sensível ao contexto para garantir a eficácia das intervenções^(1,6)^.

Dada a importância crescente dos protocolos de avaliação da linguagem na Fonoaudiologia, é essencial compreender a qualidade das medidas utilizadas e avaliar sua adequação e eficácia na prática clínica. Estes protocolos são vitais para abordar as necessidades de comunicação e comportamento em uma variedade de contextos clínicos.

Portanto, o objetivo desta revisão de escopo foi identificar e descrever os protocolos de avaliação de linguagem sob a perspectiva da ABA utilizados na fonoaudiologia. Esta revisão visa proporcionar uma compreensão abrangente dos protocolos existentes, destacando as melhores práticas e identificando possíveis lacunas na literatura, para melhorar a qualidade da avaliação e intervenção em linguagem na fonoaudiologia.

## MÉTODO

A revisão de escopo proposta foi realizada de acordo com a metodologia JBI e com base nas recomendações do Preferred Reporting Items for Systematic Reviews and Meta-Analyses (PRISMA). O protocolo foi registrado no OSF INSTITUTIONS, sob o número: 10.17605/OSF.IO/D2FMV.

### Estratégias de pesquisa

Para condução dessa revisão, adotou-se a seguinte pergunta de pesquisa: Quais são e como se caracterizam os protocolos de avaliação de linguagem baseados em ABA atualmente utilizados na Fonoaudiologia?

Os critérios para estratégia de busca foram adaptados do acrônimo PCC: População (P) - indivíduos que serão submetidos a avaliação de linguagem; Conceito (C) - Eficácia, eficiência e aplicabilidade dos protocolos de avaliação baseados em ABA; Contexto (C) – elaboração e validação de protocolos de avaliação de linguagem baseados em Análise do Comportamento Aplicada (ABA).

A busca foi realizada no dia 10 de janeiro de 2024. Realizamos buscas eletrônicas nas bases de dados LILACS, PubMed/Medline, Scopus e Web of Science, utilizando uma combinação de descritores e termos livres (Quadro 1). As referências dos estudos selecionados também foram incluídas.

**Quadro 1:** Estratégias de busca

**Base de Dados**	**Estratégia de Pesquisa**
LILACS	(“autism spectrum disorder” OR “autism” OR Children) AND (“language assessment” OR “validation study” OR “protocol” OR “language test” OR “assessment”) AND (“verbal behavior” OR “applied behavior analysis”)
PUBMED	(“autism spectrum disorder”[All Fields] OR “autism”[All Fields] OR “children”[All Fields]) AND (“language assessment”[All Fields] OR “validation study”[All Fields] OR “protocol”[All Fields] OR “language test” [All Fields] OR “assessment”[All Fields]) AND (“verbal behavior” [All Fields] OR “applied behavior analysis “[All Fields])
SCOPUS	(TITLE-ABS-KEY ((“autism spectrum disorder” OR “autism” OR “children”)) AND TITLE-ABS-KEY ((“language assessment” OR “validation study” OR “protocol” OR “language test” OR “assessment”)) AND TITLE-ABS-KEY ((“verbal behavior” OR “applied behavior analysis”)))

A busca nas bases de dados foi conduzida por dois pesquisadores de forma independente utilizando a ferramenta Rayyan, após uma calibração inicial para seleção dos artigos. A análise do material foi realizada em etapas sequenciais. Na primeira etapa, os artigos foram selecionados com base em seus títulos, levando em consideração o tema proposto. Na segunda etapa, os resumos dos artigos foram lidos para excluir aqueles que não atendiam aos critérios de elegibilidade. Na terceira etapa, os artigos que se enquadravam nos objetivos do estudo foram lidos integralmente. Na quarta etapa, foram eliminadas as referências duplicadas encontradas nas bases de dados consultadas. Em caso de divergências entre os avaliadores, um terceiro avaliador foi consultado para resolver a discordância e decidir se os artigos em questão seriam incluídos ou excluídos. Em seguida, os artigos foram analisados de acordo com a observação de variáveis como: autores, ano, base de dados, país, periódico, objetivo e desfecho. Os dados foram tabulados em planilha digital do Microsoft Excel para posterior análise, interpretação e construção de quadros descritivos.

Os dados foram analisados de forma descritiva, por meio de frequência absoluta e relativa, bem como da descrição nominal das variáveis em quadros demonstrativos, tendo em vista a quantidade de estudos selecionados, seu perfil, e níveis de evidência, que não atendem aos pré-requisitos para realização de metanálise, evidenciando a necessidade de investigação sobre o tema.

### Critérios de seleção

Incluímos estudos que investigaram o processo de validação e a aplicação dos protocolos de avaliação baseados em ABA na Fonoaudiologia; direcionados à população infantil; com foco na linguagem; contendo informações sobre as propriedades psicométricas, como confiabilidade e precisão. Não houve limite de ano e idioma. Excluímos estudos que não utilizaram protocolos de avaliação baseados em ABA, que não focaram em avaliação de linguagem, com delineamento de revisão de literatura, cartas ao editor, livros, resumos de anais de eventos científicos, artigos de opinião e artigos técnicos.

### Análise dos dados

Os artigos foram analisados de acordo com a observação de variáveis como: autores, ano, base de dados, país, periódico, objetivo e desfecho. Os dados foram tabulados em planilha digital do Microsoft Excel para posterior análise, interpretação e construção de quadros descritivos.

Os dados foram analisados de forma descritiva, por meio de frequência absoluta e relativa, bem como da descrição nominal das variáveis em quadros demonstrativos, tendo em vista a quantidade de estudos selecionados, seu perfil, e níveis de evidência, que não atendem aos pré-requisitos para realização de metanálise, evidenciando a necessidade de investigação sobre o tema.

A certeza da evidência foi analisada considerando as propriedades de medida e a qualidade metodológica, aplicando-se níveis de evidência. Este método permitirá um mapeamento abrangente e detalhado dos protocolos de avaliação ABA na fonoaudiologia, contribuindo para a otimização das práticas clínicas nesta área.

## RESULTADOS

Foram identificados em todas as bases de dados um total de 6.859 artigos, 54 na LILACS, 2.719 na PubMed/Medline, 1.537 na Scopus e 2.549 na Web of Science (WoS). Destes, 17 artigos foram selecionados pelo título, após a leitura do resumo, 12 foram excluídos por não cumprirem os critérios de inclusão: 4 eram artigos de revisão, 6 não se adequavam ao objetivo desta pesquisa e 2 estavam repetidos entre as bases de dados. Na sequência 5 foram selecionados para leitura completa do texto e posterior análise dos dados, de acordo com o fluxograma apresentado na Figura 1.

**Figura 1:** Fluxograma da estratégia de seleção dos estudos

Os Quadros 2 e 3 apontam os principais achados da literatura das variáveis referentes às características gerais dos estudos, seu objetivo e desfecho, respectivamente.

**Quadro 2:** Características gerais dos estudos que realizaram avaliação de linguagem baseada na ABA, selecionados de acordo com critérios de elegibilidade

ARTIGO	AUTOR	BASE DE DADOS	PAÍS	PERIÓDICO
1	Padilla et al.^(9)^	*PubMed*	Estados Unidos	*Journal of Autism and Developmental Disorders*
2	Barnes et al.^(10)^	*Scopus*	Estados Unidos	*Analysis Verbal Behav*
3	Sundberg e Michael^(7)^	*Scopus/WoS*	Estados Unidos	*Behavior Modification*
4	Montallana et al.^(11)^	*PubMed/Scopus*	Estados Unidos	*Journal of Autism and Developmental Disorders*
5	Myers et al.^(8)^	*WoS*	Estados Unidos	*Journal of Autism and Developmental Disorders*

Dos artigos apresentados, houve publicação entre 2001 e 2022, com um aumento na frequência de publicações a partir de 2019, (60,0%; n=3) estavam indexados na base de dados Scopus, (100,0%; n=5) são estudos realizados no Estados Unidos, e (60,0%; n=3) foram publicados na revista *Journal of Autism and Developmental Disorders* (Quadro 2).

Maioria dos estudos (n=3; 60%) dos estudos abordaram a utilização do instrumento Verbal Behavior Milestones Assessment and Placement Program (VB-MAPP), sendo dois referentes ao processo de validação e outro à sua aplicabilidade. Sundberg e Michael^(7)^ discutiram a avaliação proposta por Skinner pontuando a importância das suas dimensões para embasar a avaliação do TEA. Também foi encontrado estudo que utilizou a Avaliação de Habilidades Básicas de Linguagem e Aprendizagem - Revisada (ABLLS-R), que foi capaz de monitorar a trajetória do desenvolvimento da criança (Quadro 3).

**Quadro 3:** Descrição dos achados referentes à avaliação de linguagem baseada na ABA nos estudos selecionados

**ARTIGO**	**VARIÁVEL**	**RESULTADO**
**1**	INSTRUMENTO	Verbal Behavior Milestones Assessment and Placement Program (VB-MAPP)
	OBJETIVO	Avaliar a validade de conteúdo do VB-MAPP, incluindo a Avaliação de Habilidades E cóicas Iniciais (EESA) e a Avaliação de Barreiras
	DESFECHO	A validade do conteúdo foi moderada a forte, embora algumas áreas tivessem suporte limitado ou conflitante
**2**	INSTRUMENTO	Verbal Behavior Milestones Assessment and Placement Program (VB-MAPP)
	OBJETIVO	Examinar os efeitos do treinamento de habilidades comportamentais na administração da avaliação de marcos do VB-MAPP por profissionais da educação
	DESFECHO	O treinamento resultou em aumentos imediatos no desempenho dos profissionais
**3**	INSTRUMENTO	Análise de Skinner
	OBJETIVO	Analisar a importância da análise de Skinner sobre o comportamento verbal no tratamento de crianças com autismo
	DESFECHO	Análise do Comportamento Verbal de Skinner podem aprimorar os programas de intervenção para crianças com autismo, já beneficiadas pela Análise do Comportamento Aplicado (ABA)
**4**	INSTRUMENTO	Verbal Behavior Milestones Assessment and Placement Program (VB-MAPP)
	OBJETIVO	Explorar a consistência entre avaliadores na aplicação do VB-MAPP
	DESFECHO	Embora a Avaliação de Marcos e a Avaliação de Barreiras tenham apresentado boa e moderada confiabilidade, respectivamente, os domínios individuais de cada avaliação foram menos confiáveis.
**5**	INSTRUMENTO	Avaliação de Habilidades Básicas de Linguagem e Aprendizagem - Revisada (ABLLS-R)
	OBJETIVO	Avaliar a eficácia de um programa Medicaid estadual que fornece serviços de Intervenção Comportamental Intensiva Precoce em casa para crianças com Transtorno do Espectro Autista
	DESFECHO	Houve variação significativa nas pontuações iniciais do ABLLS-R das crianças e em suas trajetórias de crescimento, com diferenças baseadas na idade inicial, etnia e localização geográfica

## DISCUSSÃO

A avaliação da linguagem é base para as decisões clínicas. As informações coletadas direcionam as estratégias que serão utilizadas ao longo da intervenção, por isso os dados coletados devem ser funcionais e efetivos. Instrumentos de avaliação bem estruturados, validados e com boa responsividade auxiliam o processo avaliativo e possibilitam uma tomada de decisão mais segura e baseada em evidências. Por isso é importante pesquisar, conhecer e selecionar os melhores instrumentos de avaliação, tanto na clínica quanto nas pesquisas.

A adoção de protocolos efetivos de avaliação de linguagem permite a padronização das informações coletadas, a replicabilidade por outros profissionais e instituições, um monitoramento e reavaliação confiável, e consequentemente facilita a comunicação entre profissionais clínicos e pesquisadores, e até escola. Esses aspectos são importantes para indivíduos diagnosticados com TEA, acompanhados por diferentes profissionais e a longo prazo.

Apesar disso, observou-se durante as buscas que instrumentos padronizados e validados de avaliação da linguagem no TEA são escassos^(12)^. Existe a necessidade de mais investimento nos processos de validação e medidas psicométricas, ainda mais quando diz respeito à interface linguagem e ABA, tendo em vista os resultados apresentados no presente trabalho, que encontrou baixa quantidade de pesquisas sobre o tema, com apenas dois protocolos descritos, ainda não validados para a população brasileira de acordo com o preconizado, destacando a necessidade de estudos voltados à proposta e validação de instrumentos voltados à avaliação de linguagem no TEA e embasados na Análise do Comportamento Aplicada.

A ABA apresenta um papel vital no desenvolvimento de protocolos de avaliação da linguagem, especialmente no contexto de TEA. A eficácia desses protocolos reside na sua capacidade de fornecer uma compreensão detalhada das habilidades linguísticas e de comunicação de um indivíduo, permitindo intervenções personalizadas e focadas. Este artigo revisa a literatura existente sobre os protocolos de avaliação da linguagem em ABA, destacando abordagens inovadoras e práticas baseadas em evidências^(1)^.

O VB-MAPP (*Verbal Behavior Milestones Assessment and Placement Program*) é um dos protocolos apresentados nos estudos selecionados desta revisão, baseado na análise do comportamento verbal de Skinner, desenvolvido para avaliar crianças com autismo ou outros transtornos do desenvolvimento. Ele se concentra em várias áreas, incluindo marcos linguísticos, habilidades de aprendizagem e comportamento social. O programa é dividido em diferentes níveis e inclui avaliações como a de habilidades ecoicas iniciais (EESA) e uma avaliação de barreiras, que identifica obstáculos ao aprendizado e à comunicação. O VB-MAPP avalia tanto a expressão quanto a compreensão da linguagem, fornecendo um quadro abrangente do desenvolvimento linguístico da criança^(9)^.

Ele se divide em várias seções, como, a avaliação de marcos: avalia 170 marcos de desenvolvimento linguístico e de aprendizagem, divididos em 3 níveis de acordo com a idade da criança. Inclui aspectos como mando (pedido), tato (nomeação), ecoico (imitação vocal), intraverbal (conversação), habilidades de grupo, brincadeiras sociais, entre outros; avaliação de barreiras: identifica e avalia barreiras ao aprendizado, como comportamentos problemáticos, habilidades ecóicas deficientes, falta de motivação, entre outros. Essa avaliação ajuda a entender o que está impedindo a criança de aprender de maneira eficaz; avaliação de habilidades ecóicas iniciais (EESA): foca na avaliação da capacidade da criança de imitar sons e palavras, que é fundamental para o desenvolvimento da fala; avaliação de transição: destinada a crianças que estão prontas para passar de um ambiente de ensino estruturado para um mais natural, como uma sala de aula regular e o planejamento de colocação e ensino (TASK): fornece diretrizes para o desenvolvimento de planos de ensino individualizados, com base nas informações coletadas nas avaliações anteriores^(9)^.

O artigo 1, “*Evidence of Content Validity for the Verbal Behavior Milestones Assessment and Placement Program* (VB-MAPP)”, investiga a validade de conteúdo do VB-MAPP. A pesquisa envolveu um painel de especialistas que avaliaram diversos aspectos do programa, incluindo a relevância, adequação da idade e métodos de medição^(9)^.

A validade de conteúdo é um processo psicométrico recomendado pelas diretrizes de validação que observa a coerência e adequação do instrumento no que se refere ao tema, redação, formato, tarefas ou questões de um teste, instruções de aplicação resposta. elaboração dos itens, considerando-se aspectos sintáticos e semânticos que contribuam para a clareza, pertinência, coerência e abrangência dos itens, além dos aspectos operacionais^(13)^. O instrumento deve ser avaliado por experts, especialistas na área temática do instrumento, que avaliam principalmente a representatividade e relevância dos itens em relação ao desfecho proposto, por meio do cálculo do Índice de Validade de Conteúdo geral (IVC) e por Item (IVC-I) para apresentação escore de concordância entre os juízes^(13)^.

Nos resultados desta pesquisa, destaca-se a eficácia desse protocolo na avaliação de habilidades linguísticas e comunicativas. Os resultados indicaram ainda, uma validade de conteúdo de moderada a forte para o VB-MAPP, embora alguns domínios apresentassem suporte limitado ou conflitante. Isso sugere que o VB-MAPP é uma ferramenta útil, mas alguns aspectos podem precisar de refinamento. O estudo também aborda a importância de avaliações contínuas e adaptativas, essenciais para monitorar o progresso e ajustar as intervenções educacionais e terapêuticas para crianças com autismo^(9)^.

Ainda com foco no VB-MAPP, o artigo de Barnes et al.^(10)^, “*Implementing the Verbal Behavior Milestones Assessment and Placement Program (VB-MAPP): Teaching Assessment Techniques*”, examina o impacto do treinamento de habilidades comportamentais (BST) na administração do VB-MAPP por profissionais da educação. A pesquisa demonstrou que o BST resultou em melhorias imediatas no desempenho dos participantes na aplicação do VB-MAPP.

Nota-se um foco na importância do treinamento adequado para a administração eficaz do VB-MAPP, a pesquisa reforça a ideia de que profissionais bem treinados são cruciais para garantir avaliações precisas e efetivas, essenciais no desenvolvimento de planos de intervenção individualizados para crianças com autismo. O estudo também sugere que a aplicação competente do VB-MAPP pode proporcionar insights mais profundos sobre as necessidades linguísticas e de aprendizagem das crianças, direcionando intervenções mais efetivas^(10)^.

Existe uma complexidade da avaliação da linguagem em crianças com autismo e os resultados dessa pesquisa ressaltam a necessidade de uma compreensão profunda do protocolo VB-MAPP para uma aplicação eficaz. Os resultados também mostram melhorias imediatas na capacidade dos profissionais de educação em administrar o VB-MAPP após o treinamento BST, sugerindo que o treinamento direcionado pode aumentar a precisão e eficácia das avaliações. Esta constatação tem implicações significativas para a formação e desenvolvimento profissional de educadores e terapeutas que trabalham com crianças no espectro autista, enfatizando a necessidade de programas de treinamento robustos e baseados em evidências^(10)^.

Sabe-se que fatores relacionados à língua, cultura, ambiente e contexto de aplicação, características da aplicação, como interferências do aplicador, tipo de respostas, objetivas ou subjetivas, chave de resposta, tamanho do instrumento, modelo de aplicação, entrevista ou autoaplicação, podem interferir diretamente na coleta da avaliação^(14)^. Por isso é importante que ocorra a definição desses aspectos, bem como treinamento para os profissionais que farão a aplicação, como realizado no protocolo citado.

O VB-MAPP passou por outras etapas de validação. Montallana et al.^(11)^ objetivaram analisar sua confiabilidade interavaliadores do VB-MAPP. A pesquisa revelou que, embora a Avaliação de Marcos e a Avaliação de Barreiras do VB-MAPP apresentassem boa e moderada confiabilidade, respectivamente, a confiabilidade nos domínios individuais foi menos consistente.

A variabilidade na confiabilidade reforça a necessidade de treinamento adicional para os avaliadores e possíveis refinamentos no protocolo VB-MAPP. Essa discussão destaca a importância de garantir consistência e precisão nas avaliações, fundamentais para desenvolver intervenções educacionais e terapêuticas eficazes para crianças com autismo. A pesquisa também aponta para a necessidade de estudos adicionais para explorar as causas da variabilidade na confiabilidade e para otimizar o uso do VB-MAPP em diferentes contextos^(11)^.

Diferentes profissionais podem interpretar e pontuar o VB-MAPP de maneira inconsistente. Isso pode ter implicações significativas na formulação de planos educacionais e terapêuticos para crianças com autismo. Esses resultados enfatizam a necessidade de mais pesquisas para aprimorar a confiabilidade e eficácia do VB-MAPP^(11)^.

O terceiro estudo, intitulado “*The Benefits of Skinner’s Analysis of Verbal Behavior for Children With Autism*” de Mark L. Sundberg e Jack Michael^(7)^, trata-se de uma discussão teórica sobre a aplicação da análise do comportamento verbal de Skinner no contexto do autismo. O artigo sugere que esta abordagem pode enriquecer significativamente as intervenções para crianças com autismo, enfatizando a importância de compreender e ensinar diferentes operantes verbais de maneira específica e direcionada.

A análise do comportamento verbal de B.F. Skinner é uma abordagem significativa no estudo da linguagem, Skinner propõe que o comportamento verbal deve ser entendido como uma função do comportamento operante, onde a linguagem é moldada e mantida pelo ambiente. Ele identifica diferentes tipos de operantes verbais, como mando (pedido), tato (nomeação), ecoico (imitação vocal), e intraverbal (respostas verbais que não são ecos de palavras ouvidas). Essa abordagem foca na função prática da linguagem e não apenas em sua forma ou estrutura, permitindo um entendimento mais profundo sobre como as crianças com autismo aprendem e usam a linguagem em diferentes contextos^(7)^.

A análise de Skinner explora como a análise do comportamento verbal oferece uma perspectiva detalhada e funcional da linguagem, diferenciando-se de abordagens que focam apenas na forma e estrutura da linguagem. Ao entender a função da linguagem no contexto do comportamento, os terapeutas e educadores podem criar programas de intervenção mais eficazes que abordam as necessidades específicas de comunicação de cada criança com autismo. Isso pode incluir a adaptação de métodos para desenvolver habilidades de comunicação funcional e a consideração de fatores como motivação e reforço na aprendizagem da linguagem^(7)^.

O último artigo encontrado nesta revisão, “*Analysis of a Statewide Early Intervention Program for Young Children with Autism Spectrum Disorder*”, analisa a eficácia de um programa de intervenção precoce para crianças com autismo em um estado rural dos EUA. O estudo utilizou a Avaliação de Habilidades Básicas de Linguagem e Aprendizagem - Revisada (ABLLS-R) para avaliar as crianças^(8)^.

O ABLLS-R é uma ferramenta de avaliação e currículo abrangente para crianças com autismo e outros transtornos do desenvolvimento. Ele avalia uma ampla gama de habilidades linguísticas e de aprendizagem, incluindo comunicação, habilidades sociais, autocuidado, habilidades motoras e acadêmicas. O ABLLS-R é projetado para identificar deficiências específicas e desenvolver programas educacionais personalizados, baseados em princípios da ABA. É um instrumento detalhado que oferece orientações claras para medir e melhorar habilidades essenciais^(8)^.

Os resultados do estudo indicam variações significativas nas pontuações iniciais de ABLLS-R das crianças e em suas trajetórias de crescimento, sugerindo que fatores como idade de início no programa, etnia e localização geográfica podem influenciar o desenvolvimento da linguagem. Isso realça a importância de considerar esses fatores ao desenvolver e avaliar programas de intervenção. A discussão também sugere a necessidade de mais pesquisas para entender melhor como diferentes variáveis impactam o progresso de crianças com autismo em programas de intervenção precoce^(8)^.

Além disso, os resultados apontam para a importância de considerar o contexto sociocultural e ambiental na formulação de programas de intervenção, bem como a necessidade de acompanhamento longitudinal para avaliar a eficácia das intervenções ao longo do tempo. Essas descobertas são vitais para informar práticas baseadas em evidências e políticas voltadas para a educação e cuidados de crianças com autismo^(8)^.

Não foram encontrados muitos estudos que trouxessem instrumentos de avaliação baseados em ABA para avaliar a linguagem. Além disso, os estudos encontrados não contemplam a validação desses instrumentos para o português brasileiro. Existe espaço e a necessidade de mais estudos de validação de protocolos de avaliação da linguagem de crianças, especialmente daquelas com TEA.

## CONCLUSÃO

Apenas dois protocolos de avaliação de linguagem sob a perspectiva da ABA utilizados na fonoaudiologia foram encontrados nesta revisão: VB-MAPP e o ABLLS-R. Ambos não possuem estudos de validação para o português brasileiro.

Os artigos revisados demonstram a complexidade, importância e escassez dos protocolos de avaliação de linguagem em ABA, como o VB-MAPP e o ABLLS-R, na intervenção para crianças com autismo. Eles destacam ainda a necessidade de treinamento adequado para profissionais na administração dessas ferramentas e a importância de abordagens individualizadas, conforme sugerido pela análise do comportamento verbal de Skinner. A variabilidade na confiabilidade interavaliadores do VB-MAPP e as diferenças nas trajetórias de desenvolvimento linguístico observadas pelo ABLLS-R sublinham a necessidade de estratégias de avaliação adaptadas e de pesquisa contínua para aprimorar a eficácia dos protocolos de avaliação, visando uma intervenção mais eficiente de linguagem no autismo.
